# Optimized Erbium-Doped Yttrium Aluminum Garnet (Er:YAG) Laser Parameters for the Removal of Dental Ceramic Restorations

**DOI:** 10.3390/ma16175835

**Published:** 2023-08-25

**Authors:** Markus Laky, Peter Toth, Brenda Laky, Tom Vaskovich, Christoph Kurzmann, Muazzez Arslan, Mariano Nguyen, Xiaohui Rausch-Fan, Andreas Moritz, Hassan Ali Shokoohi-Tabrizi

**Affiliations:** 1 Division of Conservative Dentistry and Periodontology, University Clinic of Dentistry, Medical University of Vienna, 1090 Vienna, Austriamuazzez.arslan@meduniwien.ac.at (M.A.); mariano.nguyen@meduniwien.ac.at (M.N.); xiaohui.rausch-fan@meduniwien.ac.at (X.R.-F.); andreas.moritz@meduniwien.ac.at (A.M.); 2 Core Facility Applied Physics, Laser and CAD/CAM Technology, University Clinic of Dentistry, Medical University of Vienna, 1090 Vienna, Austria; 3 Center for Clinical Research, University Clinic of Dentistry, Medical University of Vienna, 1090 Vienna, Austria; 4 Austrian Society of Regenerative Medicine, 1010 Vienna, Austria; 5 Austrian Research Group for Regenerative and Orthopedic Medicine (AURROM), 1050 Vienna, Austria; 6 Technical Dental Laboratory, University Clinic of Dentistry, Medical University of Vienna, 1090 Vienna, Austria

**Keywords:** Er:YAG lasers, dental ceramics, absorption, transmission, restoration removal

## Abstract

Objectives: The use of lasers for debonding adhesively luted ceramic restorations is a rather recent oral laser application in dentistry. The removal of all-ceramic restorations in the mouth can often be a troublesome task. A novel method for the debonding of ceramic restorations without damaging the restorations is Er:YAG laser irradiation. The aim of this study was to evaluate the Er:YAG laser for debonding procedures of different dental ceramics and to identify appropriate laser settings. Material and methods: Lithium disilicate, zirconium-reinforced lithium silicate, feldspatic ceramic, and zirconium dioxide were investigated. Ten ceramic rectangular-shaped specimens with 1 and 2 mm thickness were produced from each material. All specimens were irradiated with four different power settings 1.5; 2.5; 3.5; 4.5 W, pulse duration 50 μs, laser repetition rate 10 Hz, time of irradiation 10 s. The transmitted energy was measured with a powermeter. Additionally the suitability of the Er:YAG laser to remove the adhesively bonded ceramic and the time until loss of retention was evaluated. Results: The transmission rate for 1 and 2 mm platelets was determined for zirconium-reinforced lithium silicate at 54.6%/35.6%, lithium disilicate at 53.2%/35.7%, zirconium dioxide at 40.6%/32.4%, and for the feldspathic ceramic at 19.4%/10.1%. For zirconium-reinforced lithium silicate and zirconium dioxide 2.5 W (250 mJ/10 Hz) was an appropriate energy level for effective debonding. Whereas for lithium disilicate and for feldspathic ceramic, 4.5 W (450 mJ/10 Hz) is required for efficient debonding. Conclusions: There are differences regarding transmission rates between ceramic types for the Er:YAG laser light and additionally depending on the type of ceramic different energy settings should be used for adequate debonding. Based on our in-vitro experiments we recommend 2.5 W for zirconium-reinforced lithium silicate and zirconium dioxide and 4.5 W for lithium disilicate and feldspatic ceramic. Transmission rates of different ceramic types and varying influences of thicknesses and bonding materials should be considered to adjust the laser parameters during laser debonding of adhesively luted all-ceramic restorations.

## 1. Introduction

Dental ceramic restorations are increasingly used in restorative dentistry because they have optical and mechanical properties corresponding to natural teeth and offer in general better esthetics compared to conventional restorations [1,2]. With the widespread use of dental ceramics, the need of removal is increasing as well [3,4]. Ceramic dental restorations (e.g., zirconium oxide) are more difficult to remove than metal alloy restorations or metal-ceramic restorations. Furthermore, the removal with standard diamond drills is time-consuming, places a burden on the patient as well as on the dentist, and results in wear of the removal devices. Additionally, the ceramic restoration is not reusable after the removal procedure with diamond drills. This is especially important in the case of an incorrect fixation. In this case, the removal with a drill also results in the destruction of the dental restoration. Unlike the conventional method, a laser-aided removal does not result in a significant change of the mechanical properties [5] and hence, could make the reuse of the restoration possible [6]. Thus, laser-aided removal is definitely an advantage in daily clinical practice, as it is possibly also a less time-consuming procedure [7].

The Er:YAG laser is a solid-state laser with a wavelength of 2940 nm. The laser light is in the infrared range of the electromagnetic spectrum and not visible to the human eye. The laser absorption transmission quotient (ATQ) depends on the properties of the material. The 2940 nm wavelength will be predominantly absorbed by water [8], which can be found in bone, dentin, and enamel [9]. The rapid local temperature increase leads to water evaporation and micro-explosions and subsequently results in hard tissue ablation. The erbium-lasers are mainly used for cavity preparation or surface conditioning. A high water content is also found in adhesive luting materials, which provide the possibility to debond ceramic restorations. Recent studies have demonstrated a predictable way of retrieving ceramic restorations with Er:YAG or Er,Cr:YSSG lasers [7,10,11,12,13,14,15].

Depending on the dental ceramic material, different ATQs could be found. The lower the ATQ the more energy will be transmitted through the dental ceramic and reach the adhesive layer. A precondition for laser debonding of the ceramic restoration is, at least in our opinion, a low ATQ. Kellesarian et al. [16] have found in their review on the laser-assisted removal of all ceramic fixed dental prostheses that the laser is a promising treatment tool but called for well-designed trials to determine the precise laser parameters and the duration of irradiation for varying ceramics. Gonzeli et al. reported that Er:YAG laser lithium disilicate crown removal was an effective and safe method, and temperature changes in the pulp chamber did not exceed the critical value of 5.5 °C [17]. Novel zircona-ceramics like 4 mol% yttria-stabilized tetragonal zirconia polycrystal (4Y-TZP) and 5Y-TZP showed laser transmission rates from 40–60% [18]. Er:YAG laser-assisted debonding may be an alternative to conventional debonding for monocrystalline ceramic brackets [19,20,21]. Er,Cr:YSGG laser irradiation with a fractional technique was effectively used for porcelain laminate veneer removal [22].

The aim of this in vitro study was to evaluate the Er:YAG laser transmission through four different dental ceramics and to assess the Er:YAG laser’s ability for debonding. The null hypothesis of our research work is that there is no difference in the debonding time in relation to the type of ceramic.

## 2. Materials and Methods

Four different types of commercially available ceramic materials were included in the experiments. The ceramic materials were produced by Ivoclar Vivadent (Schaan, Lichtenstein) and VITA Zahnfabrik (Bad Säckingen, Germany) and are shown in Table 1. Different ceramic materials were used to investigate the debonding characteristics of each material.

Five rectangular samples of 12 × 14 mm size and 1 and 2 mm thickness were produced from commercial pre-tempered CAD/CAM blanks, using the Buehler IsoMet^™^ (Buehler, Alzenau, Germany) low-speed saw with diamond-coated blades. The feldspathic, lithium disilicate, and zirconium-reinforced lithium silicate underwent final sintering according to the manufacturer’s product data-sheet. The zirconium dioxide product in its pre-sintered state was too brittle for slicing with the low-speed saw. Hence, products in an already final sintered condition were used. The thickness of the samples was measured with a micrometer screw at each corner of the rectangular samples and were polished with silicon carbide paper (grit size 500).

### 2.1. Laser Parameters

As a laser source the LightWalker^®^ AT (Fotona, Ljubljana, Slovenia) a solid-state Er:YAG (2940 nm) laser with the handpiece HR02 was used. Pulse duration 50 μs, laser repetition rate 10 Hz, and 10 s irradiation time were constant during all experiments. The laser power was changed from 1.5 W to 4.5 W with 1 W steps in between (2.5 W, 3.5 W). These power settings were chosen because they correspond to the range of normal clinical use. For power measurements, a thermal detector and the matching monitoring unit were used UP19K-15S-H5-D0/Maestro (Gentec-EO, Quebec City, QC, Canada).

### 2.2. Power Measurements

**Figure 1:**
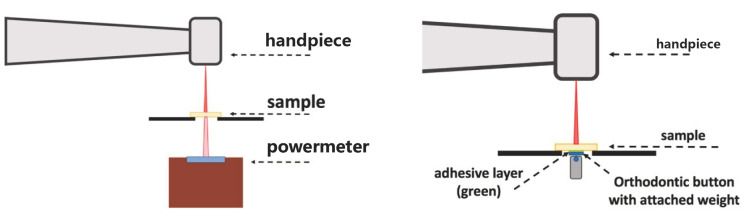
Schematic illustration of the experimental setups.

The Er:YAG laser was applied on all quadrants of each of the 1 mm- and 2 mm-samples. The laser was turned on for 10 s. However, due to a delayed reaction time of the powermeter, data were recorded for 15 s (Figure 1).

For the calculation of the transmission power, only the last 5 s of the irradiation time were used. The transmission rate was calculated as the percentage of measured transmission power from the applied laser power (100%).

Bonding of the orthodontic button with the attached weight was performed with Variolink esthetic DC (Ivoclar Vivadent, Schaan, Liechtenstein), shade neutral. The surfaces of the lithium disilicate, lithium silicate, and feldspatic ceramics were conditioned with hydrofluoric acid following the strict protocol of the manufacturer’s manual. Further surface conditioning was performed in the form of silanization with Monobond Plus (Ivoclar Vivadent, Schaan, Liechtenstein). Light polymerization was achieved with an LED lamp for 30 s (Bluephase 20i, Ivoclar Vivadent, Schaan, Liechtenstein).

Debonding was performed with the Er:YAG laser by irradiating the bonded area of an adhesively cemented orthodontic button through the ceramic material (Figure 1). The sample was fixed on the fenestrated test platelet with the bonded orthodontic button facing downwards. The button had a weight of 10 g. The distance from the handpiece to the sample surface was constantly 20 mm. The focus-to-object distance was checked and corrected before each run. The sample was then irradiated in a concentric spiral-shaped movement until the button was ‘debonded’ and dropped. The debonding was recorded with a camera and the time from the beginning of the irradiation until loss of adhesion in seconds was measured. Laser irradiation was stopped after 90 s.

## 3. Statistical Analysis

The distribution of data was assessed by visual inspection of histograms and the Kolmogorov-Smirnov-Test. Continuous data were presented as means with standard deviation as well as with median and range. Categorical variables were described as proportions and frequency counts. One-way analysis of variance (ANOVA) was used for comparison of the groups. According to the Levene F-test, if variances were or were not assumed to be equal, either the Bonferroni or the Games-Howell procedure, respectively, was used for post hoc testing. Statistical significance was reported at a *p* value of <0.05 level. All data were analyzed using SPSS software version 25 (PAWS Statistics; SPSS Inc., Chicago, IL, USA).

## 4. Results

**Figure 2:**
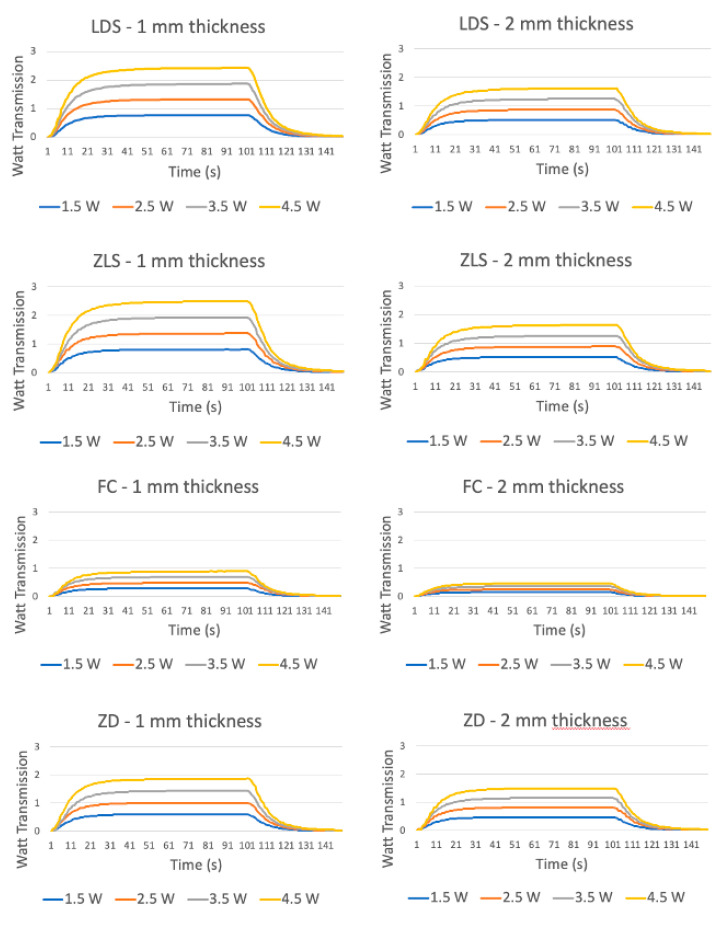
Transmission power (W) of the four materials with 1 and 2 mm thickness and an applied laser power of 1.5 W, 2.5 W, 3.5 W, and 4.5 W for 15 s/10 Hz. 4.5 watt power with a spot size of 2 mm represented a fluence of 1433 J/cm^2^.

The transmission rate of Er:YAG laser light for group I to IV is illustrated in Figure 2.

After the beginning of irradiation, the measured power quickly raised and after three to four seconds reached a constant transmission value, before dropping again after switching off the laser source. This observation was true for all tested materials and settings.

The zirconium-reinforced lithium silicate absorbed the least Er:YAG laser light, closely followed by the lithium disilicate with almost identical average values. For both lithium materials, the average transmitted power dropped by approximately one-third when increasing the thickness from 1 mm to 2 mm. The zirconium dioxide transmitted nearly a quarter less power than zirconium-reinforced lithium silicate and lithium disilicate. However, increasing the thickness of the zirconium dioxide from 1 mm to 2 mm only caused a 20% decline of average transmitted power, resulting in a similar transmission rate as lithium disilicate and zirconium-reinforced lithium silicate showed for 2 mm thickness. The feldspathic ceramic transmitted the least laser light and the increase of thickness from 1 mm to 2 mm approximately halved the transmitted power (Table 2).

The debonding time showed great variance within each group. It was generally higher in 2 mm thick samples, especially with lower power (Table 3).

**Figure 3:**
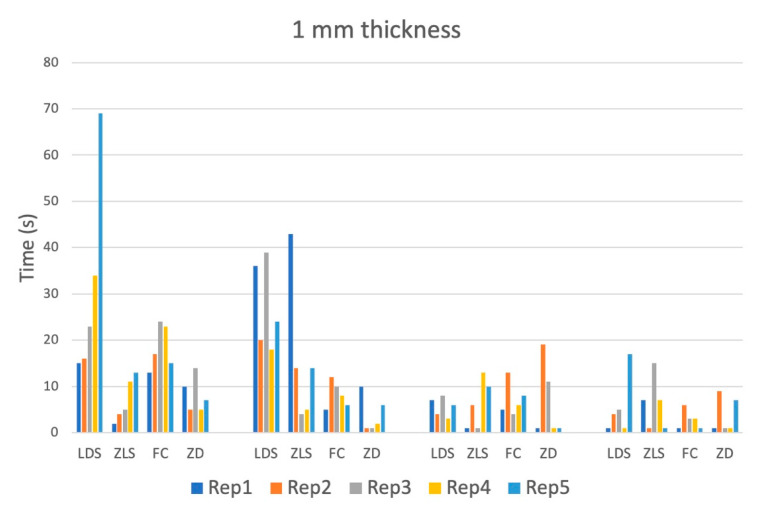
Debonding time measured until loss of adhesion (s) of all four materials for one mm thickness and all power settings. The debonding time was limited to 90 s and the test was stopped after 90 s.

**Figure 4:**
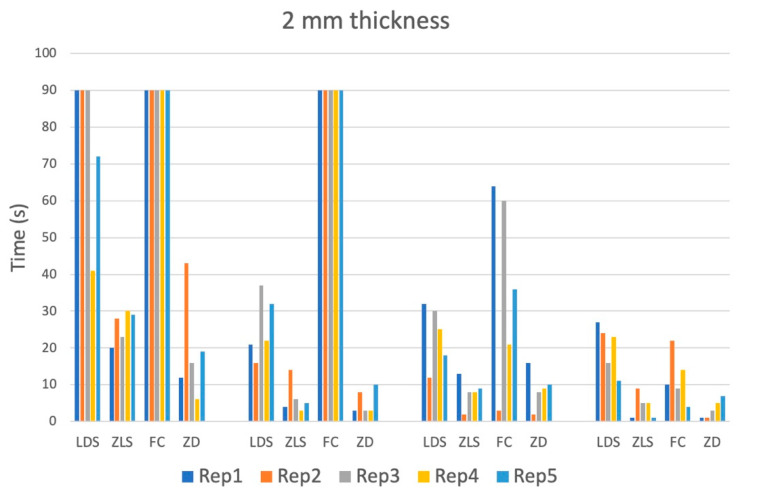
Debonding time measured until loss of adhesion (s) of all four materials for two mm thickness and all power settings. The debonding time was limited to 90 s and the test was stopped after 90 s.

Outliers on both sides of the spectrum, meaning very short as well as very long time spans until loss of adhesion, were observed (Figure 3 and Figure 4).

One-way ANOVA showed that the materials had significant effects on debonding time regarding 1 mm with 3.5 W and 4.5 W and 2 mm with all power settings (1.5 W, 2.5 W, 3.5 W, and 4.5 W). Bonferroni post hoc tests showed that there were similarities between the groups with 1 mm thickness at 3.5 W and 4.5 W (*p* > 0.999), all other comparisons show mixed results.

## 5. Discussion

Our study showed that the examined ceramic materials had different transmission rates for the Er:YAG laser irradiation. This transmission rate is an important factor for debonding treatment with a laser device. The higher the transmission rate the less time and less energy are expected to be needed for the debonding procedure with the laser. The highest transmission rate was measured for zirconium-reinforced lithium silicate (group II), followed by lithium disilicate (group I) and zirconium dioxide (group IV). The material with the lowest transmission rate was the feldspatic ceramic (group III). After passing through the ceramic material the laser energy is thereafter absorbed within the resin-cement layer because the resin-cement contains a high amount of vaporizable elements like water or residual monomers [23]. The breakdown of adhesive resin by laser irradiation is due to thermal softening, thermal ablation as well as photoablation. Thermal softening is a solely heat-dependent mechanism, ablation however is a result of resin vaporization through rapid heating and photoablation is a chemical interaction between laser light and the resin structure [9,24]. The brisk melting of the organic constituents creates large expansion forces due to the volume change of the material upon melting [25]. Temperature increases in this context may become critical if the surrounding tooth is not appropriately cooled [26].

For the 1 mm thick lithium disilicate samples Sari et al. [27] measured a transmission rate of 70% compared to the 53.2% found in this study. The different results might be explained by the handpieces used. Sari et al. used a contact handpiece (H14-C, Fotona) with a sapphire 1.3 mm laser tip. This leads to a shorter focus-to-object distance compared to the non-contact, tip-less handpiece used in this study, which might explain the higher transmission rates for lithium disilicate.

The debonding procedure was performed with the Er:YAG laser by irradiating the bonded area of an adhesively cemented orthodontic cylindrical specimen through one or two mm thick ceramic material.

Assuming that material thicknesses of more than one millimeter are to be found in the cusp area of a ceramic crown, the values of the two millimeters thick samples for debonding appear to be practicable.

To keep the debonding procedure within 10 s of laser irradiation we recommend 2.5 W (10 Hz, 250 mJ per pulse, pulse duration 50 µs) for zirconium-reinforced lithium silicate and zirconium dioxide. On the other hand, for lithium disilicate and for feldspatic ceramic 4.5 W seems to be appropriate to result in an effective debonding within a timeframe of 20 s for specimens with two mm thickness. 10 s would be needed for the debonding of a 21 mm^2^ area for zirconium-reinforced lithium silicate and zirconium dioxide. Extrapolated to the whole crown with approximately 125 mm^2^ surface area this would result in a debonding time of 60 s. For lithium disilicate and for feldspatic ceramic the time for debonding will increase to 120 s.

Application of the Er:YAG laser using the proposed parameters may be useful in the debonding of ceramic restorations, avoids damage to the restoration and helps in protecting the surrounding sound tooth substance. Our pulse duration was set to 50 µs. Shorter pulse durations were according to AlBalkhi et al. more efficient in debonding of veneeres with an Er:YAG laser [28]. Abdel Sadek et al. reported on the rebonding of laser debonded lithiumdisilicate and zirconiumoxide ceramics and found lower bonding strength [29]. Zhang et al. [5] found in a preclinical study that depending on the laser settings changes in optical properties of the lithium disilicate ceramic material occurred. It might be assumed that lower power settings might cause less damage to the removed ceramic material. In this context, our work tries to define the minimal power settings for efficient debonding of different ceramic materials. Since debonding is different for each ceramic, different laser parameters are required for effective debonding. We recommend 2.5 W for zirconium-reinforced lithium silicate and zirconium dioxide and 4.5 W for lithium disilicate and feldspatic ceramic. However, the debonding settings not only depend on the transmission rates but also on the durability of the adhesive bond of the ceramic. The null hypothesis that there is no difference in the debonding time of the different ceramic materials is rejected.

During the debonding procedure, the increase in pulpal temperature is a very important factor and also depends on the energy level used. Tissue temperature in the pulp up to 45 °C will normally not result in essential organic changes, and no irreversible tissue damage should be found. In the range of 45 to 50 °C, edema develops, and enzymatic changes occur, heating to more than 60 °C leads to coagulation and irreversible denaturation of the tissue protein [30]. The pulpal tissue of a tooth has due to the anatomic conditions compared to other tissues like the gingiva or the skin the disadvantage of a surrounding hard tissue dentinal structure without any vascularization, and the blood supply via the apex of the tooth is easily compromised. Therefore, even small areas of tissue protein denaturation may result in a subsequent necrosis of the whole pulpal area, and the pulpal tissue as a whole has to be considered especially vulnerable.

Currently a temperature rise, in the pulp above 5.5 °C is considered critical for the pulp. Such a temperature rise may result in irreversible pulpal damage [25]. Photoablative and photomechanical processes are induced with short high-power pulses. The laser irradiation leads to damage of the intracellular structures and a dissociation or ionization of the involved tissue material [30].

Rechmann et al. [25] reported a critical temperature increase during a lithium disilicate crown debonding procedure and suggested air-water cooling possibly, with water below ambient room temperature, to prevent temperature spikes and to establish sufficient heat diffusion. The thermal insulation effect of the ceramic along with the transmission rates for the laser irradiation through the ceramic could significantly exacerbate the cooling problem, as heat accumulation below the ceramic material might become problematic for the surrounding tooth tissue.

Depending on the ceramic material, prices for the individual ceramic block are between 10 and 50 € and therefore render the use of all-ceramic restoration materials cost-efficient.

A limitation of our study is the in-vitro nature of our experiments. We used a very standardized setting for the experiments with a defined thickness of the ceramic and optimal conditions for laser irradiation. The prerequisites for debonding in the mouth on a ceramic crown are significantly worse since interdental surfaces in particular are difficult to access. Therefore, our results might not be completely transferrable to the in vivo situation for a debonding procedure on a tooth. Additionally, the debonding time for a crown is only a calculated estimation from our standardized experiments. Additionally, we did not record the temperature during the debonding procedure. For future investigations, we would recommend such a temperature recording.

## 6. Conclusions

There are differences regarding transmission rates between ceramic types for the Er:YAG laser light and additionally depending on the type of ceramic different energy settings should be used for adequate debonding. Based on our in-vitro experiments we recommend 2.5 W for zirconium-reinforced lithium silicate and zirconium dioxide and 4.5 W for lithium disilicate and feldspatic ceramic. Transmission rates of different ceramic types and varying influences of thicknesses and bonding materials should be considered to adjust the laser parameters during laser debonding of adhesively luted all-ceramic restorations.

## Figures and Tables

**Table 1:** Dental ceramic types from Ivoclar Vivadent (Ivoclar Vivadent, Schaan, Liechtenstein) and VITA Zahnfabrik (Bad Säckingen, Germany).

Group	Type	Name	Company
I	Lithium disilicate (LDS)	IPS e.max^®^ CAD	Ivoclar Vivadent
II	Zirconium-reinforced lithium silicate (ZLS)	VITA SUPRINITY^®^	Vita Zahnfabrik
III	Feldspathic ceramic (FC)	VITABLOCS^®^ Mark II	Vita Zahnfabrik
IV	Zirconium dioxide (ZD)	IPS e.max^®^ ZirCAD LT	Ivoclar Vivadent

**Table 2:** Transmission rates for the different material groups; one and two mm thickness.

Power	Tickness	I LDS	II ZLS	III FC	IV ZD
1.5 W	1 mm	52.0%	53.3%	19.3%	40.0%
2.5 W	1 mm	53.2%	54.8%	19.2%	40.4%
3.5 W	1 mm	53.4%	55.1%	19.7%	40.8%
4.5 W	1 mm	54.0%	55.1%	19.5%	41.1%
1.5 W	2 mm	35.3%	35.3%	10.0%	31.3%
2.5 W	2 mm	35.2%	35.2%	10.0%	32.4%
3.5 W	2 mm	36.0%	36.0%	10.2%	32.8%
4.5 W	2 mm	36.0%	36.2%	10.2%	33.1%

**Table 3:** Comparisons of debonding time measured until loss of adhesion between all four materials for both thicknesses and all power settings.

Time (s) Mean ± SD (Median; Range)	I (LDS) N = 5	II (ZLS) N = 5	III (FC) N = 5	IV (ZD) N = 5
1 mm 1.5 W	31.4 ± 22.3 (23; 15–69)	7.0 ± 4.7 (5; 2–13)	18.4 ± 4.9 (17; 13–24)	8.2 ± 3.8 (7; 5–14)
1 mm 2.5 W	27.4 ± 9.5 (24; 18–39)	16.0 ± 15.8 (14; 4–43)	8.2 ± 2.9 (8; 5–12)	4.0 ± 3.9 (2; 1–10)
1 mm 3.5 W	5.6 ± 2.1 (6; 3–8)	6.2 ± 5.4 (6; 1–13)	7.2 ± 3.6 (6; 4–13)	6.6 ± 8.2 (1; 1–19)
1 mm 4.5 W	5.6 ± 6.6 (4; 1–17)	6.2 ± 5.8 (7; 1–15)	2.8 ± 2.0 (3; 1–6)	3.8 ± 3.9 (1; 1–9)
2 mm 1.5 W	>90	26.0 ± 4.3 (26; 20–30)	>90	19.2 ± 14.2 (16; 6–43)
2 mm 2.5 W	25.6 ± 8.6 (22; 16–37)	6.4 ± 4.4 (5; 2–13)	>90	5.4 ± 3.4 (3; 3–10)
2 mm 3.5 W	23.4 ± 8.4 (25; 12–32)	8.0 ± 4.0 (8; 2–13)	36.8 ± 25.8 (36; 3–64)	9.0 ± 5.0 (9; 2–16)
2 mm 4.5 W	20.2 ± 6.5 (23; 11–27)	4.2 ± 3.3 (5; 1–9)	11.8 ± 6.7 (10; 4–22)	3.4 ± 2.6 (3; 1–7)

## Data Availability

Data is contained within the article.
